# The impact of visual information in speech perception for individuals with hearing loss: a mini review

**DOI:** 10.3389/fpsyg.2024.1399084

**Published:** 2024-09-24

**Authors:** Ahyeon Choi, Hayoon Kim, Mina Jo, Subeen Kim, Haesun Joung, Inyong Choi, Kyogu Lee

**Affiliations:** ^1^Music and Audio Research Group, Department of Intelligence and Information, Seoul National University, Seoul, Republic of Korea; ^2^Department of Communication Sciences and Disorders, University of Iowa, Iowa City, IA, United States; ^3^Interdisciplinary Program in Artificial Intelligence, Seoul National University, Seoul, Republic of Korea; ^4^Artificial Intelligence Institute, Seoul National University, Seoul, Republic of Korea

**Keywords:** speech perception, hearing loss, cochlear implant, visual information, cross-modal plasticity, multisensory integration, age-related variation, linguistic level

## Abstract

This review examines how visual information enhances speech perception in individuals with hearing loss, focusing on the impact of age, linguistic stimuli, and specific hearing loss factors on the effectiveness of audiovisual (AV) integration. While existing studies offer varied and sometimes conflicting findings regarding the use of visual cues, our analysis shows that these key factors can distinctly shape AV speech perception outcomes. For instance, younger individuals and those who receive early intervention tend to benefit more from visual cues, particularly when linguistic complexity is lower. Additionally, languages with dense phoneme spaces demonstrate a higher dependency on visual information, underscoring the importance of tailoring rehabilitation strategies to specific linguistic contexts. By considering these influences, we highlight areas where understanding is still developing and suggest how personalized rehabilitation strategies and supportive systems could be tailored to better meet individual needs. Furthermore, this review brings attention to important aspects that warrant further investigation, aiming to refine theoretical models and contribute to more effective, customized approaches to hearing rehabilitation.

## 1 Introduction

Speech perception in individuals with hearing loss, presents a complex and multifaceted challenge. These individuals often rely on visual cues to compensate for their auditory limitations in everyday situations (Moradi et al., 2017; Tyler et al., 1997; Kaiser et al., 2003; Moody-Antonio et al., 2005; Kanekama et al., 2010). The seamless integration of auditory and visual information is particularly crucial for effective speech perception.

Previous research related to audio-visual speech perception has reported that visual cues such as lip movements, facial expressions, and gestures play a decisive role in understanding spoken language for those with hearing loss (Mitchel et al., 2023; Zhang et al., 2021; Regenbogen et al., 2012; Peelle and Sommers, 2015; Munhall et al., 2004). However, not all individuals with hearing loss benefit equally from visual information. The dependency on visual cues can significantly vary depending on factors such as the degree of hearing loss, age, and experience with communication aids like cochlear implants (CI) (Altieri and Hudock, 2014; Iler Kirk et al., 2007; Kanekama et al., 2010).

Contrary to the majority of studies that report benefits from visual information, some research shows no correlation between hearing loss and visual dependency (Lewis et al., 2018; Tye-Murray et al., 2007; Blackburn et al., 2019; Stevenson et al., 2017; Pralus et al., 2021). These studies may have been influenced by specific factors such as the subjects' aging (Tye-Murray et al., 2007), the experimental environment (Lewis et al., 2018; Blackburn et al., 2019), and the characteristics of the language stimuli (Stevenson et al., 2017; Pralus et al., 2021). Indeed, various factors, including age, experimental design, and hearing loss-related factors can contribute to the inconsistency in research outcomes (Bergeson et al., 2010).

To address these factors, this review aims to compare existing studies to gain comprehensive insights into how individuals with hearing impairments process and integrate visual cues in speech perception. Specifically, we conducted a thorough literature search using relevant keywords, such as “audiovisual” AND “speech perception” AND (“hearing impairment” OR “auditory disorder” OR “hearing defect”). The search was performed across Google Scholar and Scopus databases without restrictions on publication dates to ensure a comprehensive collection of relevant literature.

Through reviewing the extensive pool of studies collected, we identified key factors that contribute to varying outcomes in audio-visual speech perception research. These factors were categorized based on age, linguistic stimuli, and hearing loss-related factors including onset, intervention timing, and duration. Age was further classified into categories such as infants, children, adolescents, adults, and the elderly to assess the influence of developmental and aging processes. The stimuli were analyzed at different linguistic levels, including phonemes, syllables, words, and sentences, with experimental results organized into tables for detailed analysis. Additionally, cases of hearing loss were categorized based on onset, intervention timing, and duration, with interpretations of experimental results informed by these characteristics.

By evaluating various experiments from multiple perspectives according to these criteria, this review aims to provide a broad understanding of how individuals with hearing impairments process and integrate visual cues in speech perception. Ultimately, the findings from this review are expected to identify research gaps and offer valuable insights that could inform future studies in audio-visual speech perception for individuals with hearing loss.

## 2 Age-related variations in individuals with hearing loss

The first criterion we focused on is the age of individuals with hearing loss. Previous research has shown diverse age groups among participants, raising the possibility that visual information may or may not have an impact depending on age. Given the dynamic nature of language development, especially in early childhood, participant age becomes a crucial factor in integrating various sources of information. Therefore, we categorized participants into age groups encompassing infants, children, adolescents, adults, and seniors, exploring potential age-related changes in audiovisual speech perception.

Studies on infants and young children with hearing loss suggest that they become more adept at using visual information as they grow (Tona et al., 2015; Iler Kirk et al., 2007; Oryadi-Zanjani et al., 2017; Taitelbaum-Swead and Fostick, 2017). Literature frequently identifies the age of around 6 as a critical period for the initiation of sophisticated visual information utilization based on the onset of the McGurk effect and the beginning of audiovisual integration in the superior temporal sulcus (STS) (Tona et al., 2015; Iler Kirk et al., 2007; Oryadi-Zanjani et al., 2017).

Another study highlights 3.25 years as a significant age for beginning to integrate auditory and visual cues for speech perception (Holt et al., 2011). This difference can be attributed to the developmental stages of sensory integration, where basic multisensory integration starts around 3-4 years and becomes more refined by the age of 6. These developmental milestones indicate a gradual enhancement in utilizing visual information for speech perception. Compared to typically developing children, those with hearing loss are observed to rely more significantly on visual information during these early stages (Lalonde and McCreery, 2020; Oryadi-Zanjani et al., 2015, 2017; Taitelbaum-Swead and Fostick, 2017; Yamamoto et al., 2017), underscoring the role of early auditory information loss in fostering increased dependence on visual cues.

Despite the abundance of studies on infants and young children with hearing loss, research focusing specifically on adolescents is relatively scarce. While some studies include a range from infancy to adolescence, indicating that those with hearing loss may benefit more from visual information than their hearing counterparts (Tyler et al., 1997; Lamoré et al., 1998), there is a notable gap in studies dedicated exclusively to the adolescent group. This lack of research on adolescents with hearing loss suggests a need for more targeted investigation in this age group to better understand their unique challenges and benefits in utilizing visual information for speech perception.

Studies on individuals beyond the completion of language development, i.e., adults and seniors, do not pinpoint a specific age as crucial. Instead, they often analyze the influence of aging by comparing younger and older adult groups. While some studies report that older participants utilize visual information more as they age (Taitelbaum-Swead and Fostick, 2017; Puschmann et al., 2019; Hällgren et al., 2001), others suggest that the benefit of visual information remains consistent across age groups (Lasfargues-Delannoy et al., 2021; Hay-McCutcheon et al., 2005; Rigo and Lieberman, 1989). Studies specifically targeting seniors present conflicting results on how aging influences audiovisual integration, with some indicating potential benefits (Puschmann et al., 2019) and others suggesting no significant impact (Brooks et al., 2018; Tye-Murray et al., 2007). This variability underscores the importance of considering aging as a factor, dependent on experimental design and participant characteristics.

However, investigations into the neuromodulatory effects of hearing aid use and auditory training on audiovisual integration in seniors have revealed that these interventions can strengthen functional connectivity in the STS, similar to patterns observed during the developmental stages of audiovisual processing (Yu et al., 2017). While these findings are based on experiments with only two participants, they underscore the need for further research with larger sample sizes. These results highlight the necessity of considering the extent of hearing aid use and auditory training when assessing the impact of aging on audiovisual speech perception.

## 3 Linguistic level differences in individuals with hearing loss

The stimuli used in speech perception experiments are highly diverse, and changes in the linguistic level of the stimuli can significantly impact the utilization of visual information. Therefore, we categorized the experiments based on linguistic levels, such as phonemes, syllables, words, sentences, and suprasegmental elements. We then performed a comprehensive literature review, synthesizing findings from various studies to identify and describe trends in visual information utilization according to the linguistic level of the stimuli (Table 1). This approach allowed us to qualitatively assess and summarize how different linguistic levels influence the reliance on visual information in speech perception.

**Table 1:** Summary of visual salience per linguistic stimuli.

**Stimuli**	**Subgroup**	**Description**	**Visual saliency**	**Reference**
Phoneme	Labial consonants	/p/, /b/, /m/	High visual saliency due to distinct lip closure and release	(Lamoré et al., 1998; Tsao, 2019)
	Non-labial consonants	/t/, /d/, /k/, ...	Less distinct than labials but still detectable through articulatory movements	(Tye-Murray et al., 2007)
	Vowels	/a/, /e/, /i/, /o/, /u/	Sustained vowels providing moderate to high visual saliency depending on the articulatory features	(Busby et al., 1984; Lei et al., 2008)
	Syllables	–	CVC, VCV, CV, VC	High visual saliency, particularly for syllables with labial consonants	(Suess et al., 2022; Leybaert and LaSasso, 2010; Kishon-Rabin et al., 1997; Sato et al., 2020)
	Words	–	Common or nonsense words	Moderate visual saliency, influenced by the recognizability of phoneme elements, particularly labial phonemes	(Puschmann et al., 2019; Taitelbaum-Swead and Fostick, 2017; Mantokoudis et al., 2013; Tye-Murray et al., 2016; Tsao, 2019)
	Sentences	–	Simple declarative, complex sentences	Low to moderate visual saliency; effectiveness of visual information diminishes with increased linguistic complexity	(Moberly et al., 2020; Moody-Antonio et al., 2005; Hennesy et al., 2022; Tye-Murray et al., 2016; Tsao, 2019)

Phonemes, as a foundational linguistic element, is particularly influenced by visual information, leading to notable improvements in speech perception. Researches consistently demonstrate that visual information significantly enhances phoneme perception for individuals with hearing loss (Tona et al., 2015; Huyse et al., 2013; Rouger et al., 2008). Both vowel identification (Busby et al., 1984) and consonant identification (Tye-Murray et al., 2007) experiments performed better in the audiovisual than audio-only or visual-only conditions. Researchers differ in their claims about whether vowels or phonemes are generally more visually salient to further aid audiovisual integration. Some studies argue that vowels benefit more from visual cues due to sustained articulatory features and clearer formant structure (Lei et al., 2008). In contrast, others assert consonants are more salient due to distinct visual cues (Van Soeren, 2023; Moradi et al., 2017). This discrepancy may arise from the specific phonemes selected for the experiments. In any case, studies consistently agree that labial consonants (e.g., /p/, /b/, /m/) are the most visually distinctive stimuli compared to vowels and coronal phonemes due to distinct articulatory features such as lip closure and release, which are easily detectable by lip-reading (Lamoré et al., 1998; Tsao, 2019). In the same vein, Rouger et al. (2008) reported that French-speaking CI users placed greater reliance on visual information for labial phonemes, particularly under conditions of auditory uncertainty.

At the syllable level, individuals with hearing loss also benefit from audiovisual (AV) integration, facilitating speech perception (Leybaert and LaSasso, 2010; Kishon-Rabin et al., 1997; Sato et al., 2020). Studies reveal that labial syllables containing labial consonants provide greater visual cues, enhancing perceptual accuracy. For instance, a study on auditory-visual integration with hearing loss focusing on consonant recognition training highlighted overall improvements across all consonants, with significant gains observed in labial consonants within nonsense syllables (Grant et al., 1998). These findings align with those of Tona et al. (2015) and Yamamoto et al. (2017), who observed that Japanese children with cochlear implants (CIs) showed improved recognition of labial syllables (/ba/, /pa/) due to the distinct visual information provided by lip movements, compared to non-labial syllables (/da/, /ga/, /ka/, /ta/). Similarly, Suess et al. (2022) reported that bilabial syllables contribute more to the recognition of hearing-impaired, indicating that the informativeness of each viseme category varies subtly.

The influence of visual information on word recognition presents mixed results, though it generally has a positive impact. Tye-Murray et al. (2007) suggests that the recognizability of a word can be predicted based on the independent recognition of its phoneme elements as transmitted components. Bernstein et al. (2000) examined how certain phonological features influence word recognition visually, pointing out that words with round and coronal phonological traits were more effectively transmitted than those with continuant or voicing traits. This suggests that labial phonemes play a crucial role in transmitting speech and lexical content in an audiovisual context. Accordingly, studies emphasize the positive impact of visual information on word recognition (Puschmann et al., 2019; Taitelbaum-Swead and Fostick, 2017; Mantokoudis et al., 2013). However, Altieri and Hudock (2014) have argued that audiovisual integration does not provide significant benefits over auditory-only input in word recognition, challenging the general assumption of the advantages of visual information.

Sentence recognition demonstrates a consistently lower visual information effectiveness than stimuli of smaller units. Some studies show increased reliance on visual enhancement during sentence recognition for individuals with severe hearing impairment (Moberly et al., 2020; Moody-Antonio et al., 2005; Hennesy et al., 2022). However, others suggest that the hypothesis that hearing impairment enhances lipreading ability is supported at the word level but not at the sentence level (Tye-Murray et al., 2007). Similarly, Tsao (2019) highlighted audiovisual benefits at the word level, but it remained unclear whether these benefits extend to larger linguistic units, such as sentences. Words and sentences involve additional cognitive processes like lexical access and syntactic parsing, which can either enhance or diminish the role of visual information depending on the context (Jackendoff, 2002). For instance, in sentence-level stimuli, visual cues related to prosody and facial expressions become more relevant, especially for conveying emotions or stress patterns, which are crucial for understanding the overall message (Buchan et al., 2008).

Apart from a trend in segmental units such as phoneme, syllable, word, and sentence, results indicate that for suprasegmental elements, such as intonation and stress within utterance components, visual information does not provide substantial aid (Stevenson et al., 2017; Tsao, 2019). According to Stevenson et al. (2017), CI users derive meaningful visual assistance in phonemic perception and, to a certain extent, for word and sentence recognition tasks. Yet, this assistance is not extended to suprasegmental information. Furthermore, a study on Chinese users (Tsao, 2019) exploring the enhancement of speech perception through AV integration does not demonstrate a discernible contribution of visual elements to tone differentiation.

Overall, smaller linguistic units consistently show benefits from visual information for individuals with hearing impairment, who are strongly affected by labial phonological features. However, as linguistic units increase in complexity, the influence of visual information becomes less pronounced. For smaller units like phonemes and syllables, listeners detect speech by assigning the signal more directly to a phonetic category. In contrast, sentence recognition requires listeners to access phonetic and lexical representations and make corresponding decisions.

## 4 Hearing loss factors: onset, intervention timing, and cross-modal plasticity

In the preceding two chapters, we reviewed how factors such as age and linguistic level can lead to varying outcomes in the utilization of visual information for speech perception among individuals with hearing loss. Another crucial factor to consider is the characteristics related to hearing loss itself. Specifically, we aim to compare the utilization of visual information based on the onset of hearing loss and the timing of cochlear implant (CI) and hearing aid (HA) interventions.

The timing of hearing loss onset and the point at which auditory devices are worn can significantly influence how individuals perceive speech, moderating the extent to which visual information is utilized (Tyler et al., 1997; Tona et al., 2015; Colmenero et al., 2004; Bavelier et al., 2006; Stevens and Neville, 2006). Additionally, since the onset of hearing loss and the timing of intervention can affect cross-modal plasticity (Kral and Sharma, 2023; Buckley and Tobey, 2011), it is important to examine how these factors impact the utilization of visual information. Therefore, this chapter aims to review (1) the onset and intervention timing of hearing loss, and (2) cross-modal plasticity on the utilization of visual information for speech perception among individuals with hearing loss.

### 4.1 Three cases based on the onset and intervention timing of hearing loss

**Figure 1:**
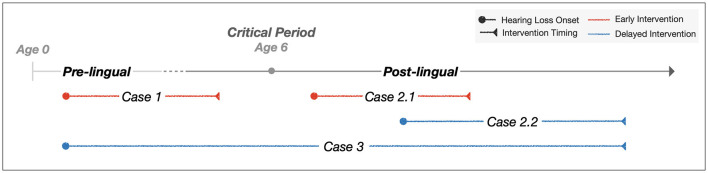
Schematic representation of hearing loss onset and intervention timing across pre-lingual and post-lingual periods.

To better understand the impact of intervention timing on speech perception outcomes in individuals with hearing loss, we have created a schematic representation of hearing loss onset and intervention timing across pre-lingual and post-lingual periods. Figure 1 presents three case categories based on these factors. The pre-lingual period, typically occurring before age 6, encompasses the critical period for language development. Case 1 involves pre-lingual hearing loss with corresponding pre-lingual intervention. Case 2 represents post-lingual hearing loss with post-lingual intervention, further divided into Case 2.1 (shorter duration before intervention) and Case 2.2 (longer duration). Case 3 illustrates pre-lingual hearing loss with delayed intervention after language acquisition.

The onset of hearing loss, whether it occurs before or after language acquisition, plays a crucial role in shaping the development of cognitive and perceptual abilities. A study by AuerJr and Bernstein (2007) highlights that individuals with early-onset hearing loss exhibit superior speech-reading abilities compared to those with normal hearing. This finding suggests that these individuals may develop compensatory strategies that enhance their capacity to interpret speech through visual cues, such as lip movements and facial expressions.

Intervention timing is closely related to the duration of hearing loss, with early intervention corresponding to a “shorter duration” (e.g., Case 1, 2.1) and delayed intervention to a longer duration (e.g., Case 2.2, 3). According to research by Gilley et al. (2010), early intervention leads to better audiovisual (AV) integration abilities, even when cochlear implants are worn for the same duration. This suggests that a shorter duration of hearing loss before intervention results in better cognitive outcomes in AV speech processing.

Moreover, in the case of early intervention, particularly for early-onset hearing loss (e.g., Case 1), Jerger et al. (2020) compared children with pre-lingual hearing loss who began using hearing aids around age 2 to their normal-hearing peers. The study found that speech detection and attention significantly improved when visual information was added to auditory-only stimuli. Building on the McGurk effect, Tona et al. (2015) demonstrated that children who received cochlear implants before age 3 utilized visual information to supplement auditory information. Similarly, Iler Kirk et al. (2007) suggested that children with cochlear implants could significantly enhance word recognition through audiovisual (AV) integration facilitated by lip reading.

Comparing intervention timings for individuals with the same onset of hearing loss reveals significant differences in outcomes. Both cases involve early-onset hearing loss, with Case 1 receiving early intervention and Case 3 undergoing intervention later in life. Case 3 involves individuals who experienced pre-lingual deafness but received intervention only after acquiring some language skills, indicating a longer duration of hearing loss. Although such cases are relatively rare and have received limited research attention, Moody-Antonio et al. (2005) provide evidence that some adults with congenital hearing impairment show significant improvements in speech perception after receiving intervention. This suggests that even without early auditory language experience, individuals can integrate auditory information from the intervention with visual language cues, demonstrating potential for enhanced speech perception despite missing the 'critical period' for early intervention.

However, differences in the use of visual information due to the timing of hearing loss and intervention between Case 1 and Case 3 are noteworthy. Research indicates that earlier intervention generally leads to better outcomes compared to later intervention. For instance, Tyler et al. (1997) found higher scores in tasks like vowel recognition and word comprehension when intervention occurred before age 5. Kral et al. (2019) suggest that intervening before age 3 is most effective for language acquisition. Additionally, Kanekama et al. (2010) highlight that intervention within 5 years of onset is particularly beneficial.

Comparing cases with the same onset and intervention timing but differing durations highlights the impact of the duration of hearing loss on language recognition outcomes. Case 2.1 experienced intervention after a relatively short duration of hearing loss, while Case 2.2 had a longer duration before intervention. The decision to categorize Case 2.1 and Case 2.2 based on duration stems from the limitations in existing research. In the study by Baskent and Bazo (2011), the wide age range of participants complicates the ability to isolate the effects of duration due to cognitive decline and age-related factors. Additionally, many studies do not provide specific details about the duration of hearing loss, making it difficult to draw clear conclusions about the effects of different durations within the post-lingual hearing loss group.

Bernhard et al. (2021) suggest that language recognition, assessed through sentence and single-word identification, shows a negative correlation with the duration of hearing loss before cochlear implantation. Lee et al. (2021) confirmed that the duration of hearing loss significantly negatively impacts both audio-only and audio-visual speech recognition. These findings indicate that even with similar onset and intervention timing, a shorter duration of hearing loss (Case 2.1) is more advantageous for language recognition compared to a longer duration (Case 2.2).

In this section, we have analyzed interventions broadly, including both cochlear implants (CI) and hearing aids (HA). However, further detailed comparative analysis of each type of assistive device is warranted. Additionally, it would be valuable to explore not only the duration of hearing loss but also the impact of the length of time using hearing aids or cochlear implants on training outcomes. Furthermore, to address the research gaps identified in the three cases discussed, there is a need for targeted additional studies for each case to better understand their specific contexts and outcomes.

### 4.2 Cross-modal plasticity with hearing loss patient

Cross-modal plasticity is commonly observed in individuals with hearing impairments and continues to be a factor even after cochlear implantation (Kral and Sharma, 2023; Lomber et al., 2010; Stropahl and Debener, 2017; Bavelier and Hirshorn, 2010; Campbell and Sharma, 2016). when individuals with hearing impairments are deprived of auditory input, much of the auditory cortex is commandeered by visual processing to compensate for the lack of auditory input. This cross-modal compensatory reorganization, where increased emphasis is placed on processing visual information, is a crucial aspect of adaptation in those with hearing impairments and has been pointed out as an important factor in the individual differences in their audio visual speech perception.

Previous studies have often shown that cross-modality may disrupt the integrative functions of the auditory cortex, potentially hindering speech perception adaptation in CI users (Liang et al., 2017). However, plasticity does not refer to a fixed or unchanging property, but rather to the ability to change for adaptation. In other words, the reorganization of the auditory cortex, which has been largely occupied to compensate for adaptation and deprivation before cochlear implant (CI) implantation, can change again after implantation. For example, a study on cross-modal plasticity before and after implantation due to visual speech revealed that cross-modal activation in the auditory regions after implantation is associated with better speech understanding, and that the simultaneous development of activation in the auditory cortex by both visual and auditory speech provides adaptive benefits (Anderson et al., 2017). Another study demonstrated that the imbalanced cross-modal activity in the auditory cortex observed in individuals with hearing impairments returned to normal levels comparable to those of the control group after cochlear implant (CI) implantation. Additionally, the activation of Broca's area, which showed lower activity during speechreading compared to that of normal individuals, increased after CI implantation. Broca's area is known as a brain region that contributes to speech production. This suggests that the recovery of auditory input through CI can plastically adjust the imbalance in the auditory cortex (Rouger et al., 2012).

There is also research suggesting that the use of visual cues varies depending on the proficiency of CI users. For example, Kelly N. Jahn observed that proficient CI users process visual cues differently than non-proficient users, supported by brain imaging studies showing distinct patterns of brain activation in response to visual cues (Jahn et al., 2017; Doucet et al., 2006; Sharma et al., 2015). However, the differences in visual cue utilization patterns suggest a complex relationship with cross-modality, but the exact correlation remains unclear. This is a topic that will require further exploration and research in the future.

The divergence in findings regarding the changes and adaptiveness of cross-modality may be attributed to various factors such as the timing of hearing loss onset–whether it is prelingual or postlingual, the duration of hearing impairment-how long one has experienced hearlong loss. Although previous studies have categorized participants based on the onset of hearing loss, they have observed cross-modality in both groups. Variables like age, language, and the nature of the tasks employed in the studies may also contribute to these varied results. To unravel this complexity, future research must refine the control of these variables. While there is extensive literature on the cross-modality of CI users, particularly concerning the clinical impact of CI on speech perception outcomes, more in-depth studies are needed on the long-term evolution of cross-modality after cochlear implantation and its influence on the integrated perception of speech. Additionally, with advancements in cochlear implant technology, the time required to achieve adaptive and balanced cross-modality may decrease. This remains a promising topic for further exploration and should be actively discussed in future research.

## 5 Discussion

This review has explored the intricate dynamics of AV speech perception in individuals with hearing loss, with a particular focus on the influences of age, stimuli diversity, and hearing loss factors. Our qualitative analysis reveals that these factors significantly modulate the reliance on and the efficacy of visual information in supplementing auditory cues for speech perception. Building on these findings, we discuss the research gaps related to each factor, propose directions for future studies, and highlight the implications and potential applications of our results.

### 5.1 Age-related variation

Based on the review of literature across different age groups, it is evident that early life stages of language and cognitive development, as well as the later stages of aging, are particularly critical periods that can significantly influence the use of visual information in speech perception. Specifically regarding aging, considering the variability in results due to task differences and sensory loss (Brooks et al., 2018), additional research that controls for task and age-related characteristics of participants in a consistent manner across age groups is warranted. Furthermore, given the lack of literature on adolescents, recruiting participants from all age groups–infants, children, adolescents, adults, and the elderly–and measuring AV speech perception using the same experimental design would provide clearer insights into age-related effects. Additionally, considering the neuromodulatory effects of aging on sensory integration in speech perception (Yu et al., 2017), there is a need for age-tailored rehabilitation strategies for individuals with hearing loss.

### 5.2 Linguistic diversity

The variability in response to different linguistic stimuli highlights the complexity of AV speech perception. Labial consonants, for example, are more readily enhanced by visual cues, pointing to the significant role of visually salient speech features in improving perception. However, the effectiveness of AV integration in speech perception is influenced by language-specific viseme configurations, with languages having denser phoneme spaces (e.g., English, French) showing a higher visual dependency. Investigating the efficacy of such tailored interventions across diverse linguistic contexts remains an essential avenue for future research. Also, there is a need for further comparative analysis across linguistic levels, particularly examining the relationship between phoneme recognition and higher linguistic units, as discussed in studies such as Lamoré et al. (1998) to understand how visual information contributes to speech perception as linguistic complexity increases. Moreover, this finding calls for a nuanced approach to auditory rehabilitation, suggesting tailored AV training programs emphasizing visually salient features to enhance speech perception for hearing loss such as Schumann and Ross (2022), or even further, strengthening the connection between these phonetic and lexical representations (Tye-Murray et al., 2016).

### 5.3 Hearing loss factors

This review has highlighted the critical influence of the onset of hearing loss and the timing of intervention on the utilization of visual information in speech perception. Early intervention, particularly in cases of pre-lingual hearing loss, significantly enhances speech perception by optimizing the use of visual cues. In contrast, later interventions, whether in pre-lingual or post-lingual cases, show varied outcomes influenced by factors such as cognitive decline and the duration of hearing loss. A key factor in these processes is cross-modal plasticity, where the auditory cortex is reorganized to process visual information in response to auditory deprivation. Although this reorganization can initially support speech perception through enhanced visual reliance, its persistence post-cochlear implantation may disrupt the balance between auditory and visual inputs. Therefore, understanding the long-term effects of cross-modal plasticity, particularly how it evolves after CI implantation, is crucial. Future research should investigate the impact of the duration of hearing loss before intervention and how individual differences in cross-modal plasticity influence speech perception outcomes. Additionally, the development of adaptive technologies and tailored rehabilitation strategies that consider these factors could significantly improve audiovisual integration for CI users, ultimately enhancing their speech perception abilities in diverse listening environments.

### 5.4 General discussion

Moving forward, future research should prioritize the practical applications of visual information in hearing rehabilitation. Developing guidelines for personalized rehabilitation plans that consider age, type of stimuli, and specific characteristics of hearing loss will be crucial. One promising approach involves the integration of AI-assisted training systems, which can customize learning paths based on the user's individual characteristics, such as age, degree of hearing loss, and the use of assistive devices. These systems can provide real-time feedback and adaptive learning by analyzing how effectively a user utilizes visual cues during speech perception tasks. For instance, if a user struggles with specific phonemes or words, the AI can detect this and adjust the training regimen, offering additional practice or emphasizing visual cues more heavily to optimize learning outcomes.

Moreover, AI systems can support the learning of various linguistic and phonetic features, tailoring exercises to the user's linguistic background. For example, in languages with dense phoneme spaces like English, the system could focus more on tasks that rely heavily on visual cues, whereas in vowel-centric languages, it could emphasize the visual recognition of vowels. Additionally, these systems can strengthen the integration of visual and auditory information by using training methods based on phenomena like the McGurk effect, helping users more effectively combine these sensory inputs. The long-term tracking and analysis of a user's progress would allow for continuous refinement of personalized learning strategies, which is particularly beneficial for those receiving later-stage interventions.

By focusing on these areas, we can create more effective, tailored interventions that fully leverage both auditory and visual cues. Such advancements will not only improve speech perception and communication abilities but also significantly enhance the quality of life for those affected by hearing loss. Through this review and discussions, we have gained a deeper understanding of how visual information influences speech perception in individuals with hearing loss, particularly in relation to the critical roles of age, linguistic diversity, and the timing of hearing loss. By integrating these variables into future studies on audiovisual speech perception, we can refine theoretical models, bridge gaps in current research, and enhance the predictive power of future investigations. The ongoing exploration of these factors is essential for advancing our knowledge of sensory integration processes and developing more holistic and personalized approaches to hearing rehabilitation.
